# General public awareness, knowledge and attitude toward COVID-19 infection and prevention: a cross-sectional study from Pakistan

**DOI:** 10.12688/f1000research.52692.1

**Published:** 2021-09-21

**Authors:** Beenish Fatima Alam, Abdullah A. Almojaibel, Khalid Aziz Ansari, Mohammad Haroon, Sara Noreen, Saman Tauqir, Khalid Almas, Faraz A. Farooqi, Saqib Ali

**Affiliations:** 1 Department of Oral Biology, Bahria University Medical and Dental College, Karachi, Pakistan; 2 Respiratory Care Department, College of Applied Medical Sciences, Imam Abdulrahman Bin Faisal University, Dammam, Saudi Arabia; 3 Department of Medicine, Khyber Teaching Hospital, Peshawar, Pakistan; 4 Department of Physiology, Kabir Medical College, Gandhara University, Peshawar, Pakistan; 5 Department of Preventive Dental Sciences, College of Dentistry, Imam Abdulrahman Bin Faisal University, Dammam, Saudi Arabia; 6 Department of Dental Education, College of Dentistry, Imam Abdulrahman Bin Faisal University, Dammam, Saudi Arabia; 7 Department of Biomedical Dental Sciences, College of Dentistry, Imam Abdulrahman Bin Faisal University, Dammam, Saudi Arabia

**Keywords:** COVID-19, Awareness, Knowledge, Perception, Pakistan, Pandemic

## Abstract

**Background:** The aim of this study is to evaluate the knowledge, perceptions, and attitude of the public in Pakistan (using social media) towards COVID-19.

**Methods: **A cross-sectional study was conducted amongst 1120 individuals nationwide. A self-developed, pre-tested questionnaire was used that comprised of sections covering demographic characteristics, medical history, hygiene awareness, COVID-19-related knowledge, and learning attitude. Descriptive statistics were used for frequencies, percentages, averages and standard deviations. Inferential statistics were done using the Student’s t-test and ANOVA.

**Results:** The average age of participants was 31 years (range 18-60 years). In total 56 individuals (5%) had completed primary or secondary school education; 448 (40%) were employed (working from home) and 60% were jobless due to the COVID-19 crisis. Almost all the study subjects (1030 (92%)) were washing their hands multiple times a day. A total of 83% had awareness regarding quarantine time, 82% used face masks whenever they left their homes, 98% were aware of the origin of the disease, and 70% had knowledge regarding the most common symptoms of COVID-19.

**Conclusion:** It can be concluded from the current study that female participants had higher level of education, and more awareness regarding the coronavirus. The majority of the participants followed proper hand washing regimes and washed their faces. Further knowledge and awareness should be promoted.

## Introduction

The first case of COVID-19 was identified within the Wuhan city of China in December 2019. ^1^ By February, a tremendous increase in the number of cases and number of deaths due to Covid-19 started being reported. Due to the rapid spread of infection from China to different countries, and with the number of cases exceeding the cases reported by the Chinese government, COVID-19 was declared a global pandemic by the World Health Organisation in March 2020. ^2^^,^^3^ This epidemic has now affected more than 200 different countries across the globe. ^4^

COVID-19 is a highly contagious infection that spreads through human to human contact causing serious health problems within communities. ^5^ Currently very little scientific information is available regarding this novel virus. However, it has been identified as an enveloped RNA virus that is further categorised into alpha, beta, gamma and delta. ^6^ Depending upon the immune system of an individual it can cause symptoms ranging from mild to severe. Symptoms identified comprise of fever, cough, difficulty in breathing, loss of smell, while in extreme cases can lead to pneumonia, multi-organ failure and death. ^7^ The rapid spread of this infection is principally through respiratory droplets of 5-10 μm in diameter, spread through the mouth or nose, when an infected person coughs or sneezes. ^8^ A study conducted in Singapore has identified that these droplets can be transmitted across a distance of 4.5 metres. ^9^^,^^10^ Moreover symptoms can be identified within a day or two after acquiring infection, extending up to 14 days. ^11^

The first positive case of COVID-19 in Pakistan was identified on 26 ^th^ February 2020. In order to control the spread of the disease, on 23 ^rd^ March 2020 a complete lockdown was imposed throughout the country. This included closure of all the educational organizations that comprised of schools, colleges, and universities along with religious schools. ^12^ All ceremonies and religious gatherings in any form were adjourned to prevent the spread of infection to the general public. ^13^ It was highlighted that elderly people and individuals having systemic disease, such as heart disease, diabetes, cancer and respiratory disease, are at high risk of acquiring this infection, while children appear to be less susceptible towards this disease. Proper protocol must be followed to prevent the spread of this disease. ^14^

Currently there is a secondary wave or surge reported from many parts of the world; therefore it is of great importance for the general public to have appropriate awareness regarding the signs and symptoms and the causative factors of this disease and preventive protocols that need to be followed, including social distancing, following proper hand hygiene protocol and use of face masks. As Pakistan is a developing country with limited resources, the country has introduced different strategies to increase the awareness of the general public. Therefore, this study was undertaken to analyse the level of awareness, knowledge and practices of the public of Pakistan using an online survey regarding COVID-19 and various measures to be undertaken to prevent the spread of this disease.

## Methods

### Study design

This cross-sectional study was undertaken among the public of Pakistan using and online questionnaire. The questionnaire was sent to the general public through various social platforms (Whatsapp, Twitter, Emails, Facebook messengers).

### Participants

Individuals of both genders, within the age group of 18 to 60 years of age, of all education levels were included in this survey. Informed consent was taken from all the participants before asking them to complete the survey. Responses were kept anonymous to maintain the confidentiality of the participants. Five to seven minutes were required to complete the survey.

#### Data collection

Due to complete closure of public places, an online survey was conducted using Google forms. Data was collected from May through to June 2020.

The questionnaire used for this study was developed by the authors and adapted from Ali et al. ^29^ Before collecting the data, pilot testing of the questionnaire was carried out among 20 individuals (selected from social media groups within the community) to test the reliability and validity of the questionnaire. Following their responses, necessary corrections (simplification of terms) were carried out.

The questionnaire utilised for this study was divided into multiple sections as follows: demographic characteristics (gender, education level, income earned, job status); section related to health (presence of any systemic disease and smoking habits); hygiene awareness (frequency of washing hands and face, type of soap used, duration of hand washing, frequency of touching face); COVID-19 related knowledge (origin, symptoms and transmission route for COVID-19); measures taken for prevention against COVID-19 (knowledge regarding usage, indications and different types of face masks); and lastly learning attitudes regarding COVID-19. There were 7 main questions covering COVID-19 knowledge. Three of which had multiple correct answers, each correct answer was given 1 point and wrong answer counted as 0. Total maximum knowledge score was 16.

### Statistical analysis

SPSS version 22 was used for data analysis. Descriptive statistics including frequencies, percentages, means and standard deviations were used to determine demographical information, health history and knowledge related questions. Student’s t-test and ANOVA were used for comparing knowledge score with demographical information and awareness related responses. Multiple comparisons were done between demographics of participants in case of significant relation found. P-value <0.05 was considered as statistically significant. Univariate and multivariate regression analysis was used to investigate the factor associated with knowledge score. P-value 0.10 was considered as cut off for significant value for factors in univariate analysis.

### Ethics approval and consent to participate

Ethical permission was obtained from the Bahria Medical and Dental College Ethics Review committee to conduct the study (reference number ERC 55/2020). A participation consent statement was added before the survey as follows: “Your participation in this study is completely voluntary. There are no foreseeable risks associated with this project. However, if you feel uncomfortable answering any questions, you can withdraw from the survey at any point”. Participation in the survey was therefore taken as consent to participate.

## Results

A total of 1,120 participants completed the questionnaire. Table 1 shows the demographic characteristics and health history of participants. Average age of participants was 31 years (ranged from 18 to 60 years). Of the sample, 52% were in age range of 30 to 45 years. Most of the participants were male (604; 54%); there were 516 (46%) women. Almost half of the participants had postgraduate degrees (526; 47%), 470 (42%) were college graduates and 6% had doctorates. Only 56 (5%) individuals had completed primary or secondary education and could read or write. A total of 448 (40%) individuals were employed (working from home), while more than half (60%) were jobless due to the COVID-19 crises. Most belonged to the lower income group ranging from 10,000 to 30,000 PKR. A total of 862 (77%) of the participants were free from any allergy or respiratory diseases, with only 10% suffering from seasonal allergies. Similarly, 840 (75%) participants expressed themselves as fit and medically healthy.

**Table 1.** Demographic characteristics of the participants.

Demographic characteristics	n	%
**Gender**		
Male	604	54
Female	516	46
**Age in years**		
15-30	482	43.0
30-45	582	52.0
45-60	56	5.0
**Education level**		
Primary/Secondary	56	5
College Graduate	470	42
Postgraduate	526	47
Doctorate	68	6
**Working status**		
Yes (working from home)	448	40
No	672	60
**Monthly income**		
Less than 30K	414.4	37
30K to 50K	403.2	36
More than 50K	302.4	27
**Smoking status**		
Yes	145.6	13
No	862.4	77
Occasional smoker	78.4	7
Past smoker	33.6	3
**Health background**		
*Respiratory disease or any allergy*		
Yes	145.6	13
No	862.4	77
Seasonal/medicinal allergy	112	10
*Underlying medical condition/systemic diseases*		
Fit and healthy	840	75
Heart disease	11.2	1
Hypertension	67.2	6
Arthritis	22.4	2
Any other disease	179.2	16

**Figure 1.**
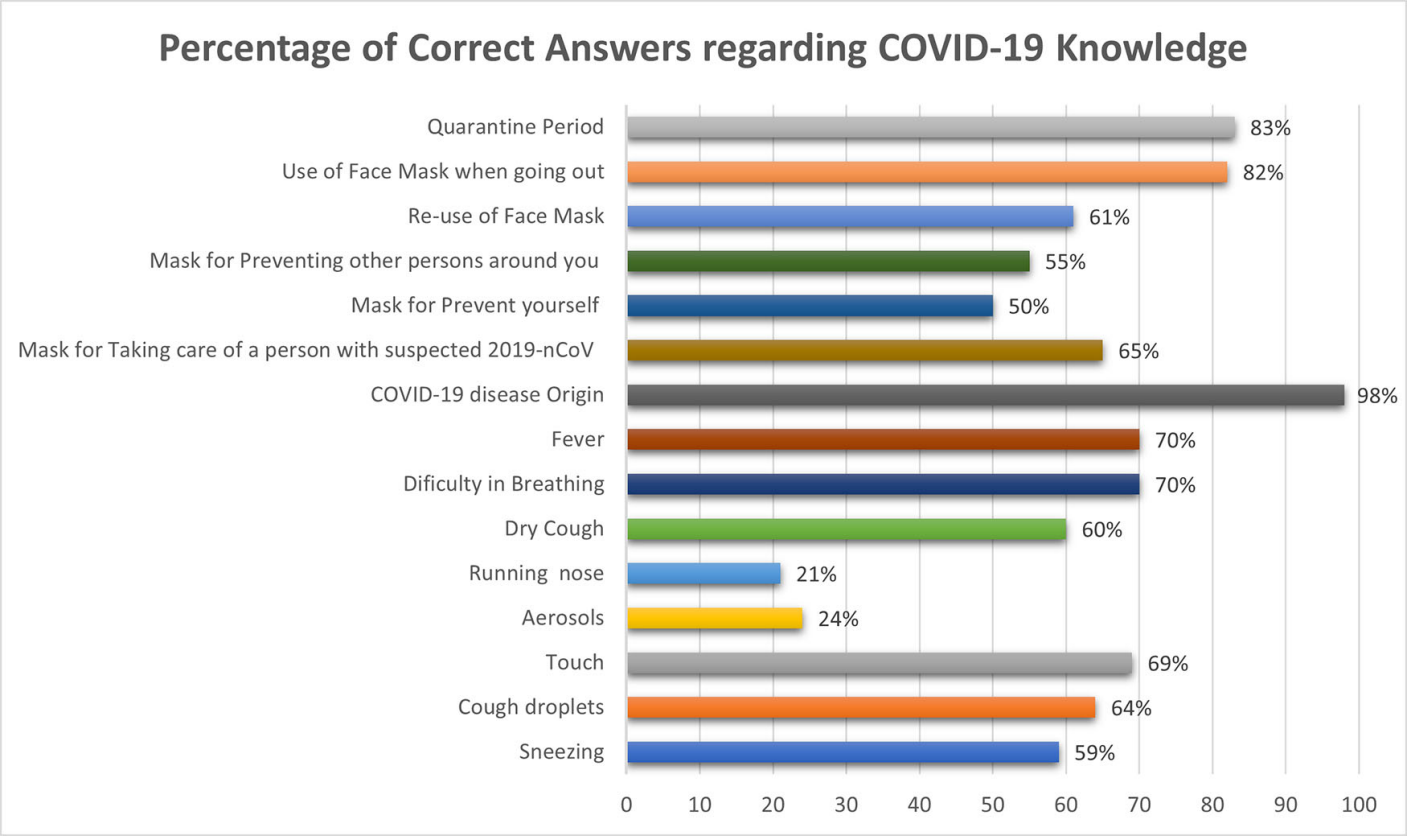
Percentage of correct answers regarding COVID-19 knowledge.

Most of the participants (70%) claimed that they have good knowledge of novel COVID-19 disease. Mean knowledge score was 9.48±2.88 out of 16, which can be considered as good knowledge about the disease. Figure 1 shows the correct answer percentages of knowledge questions asked: 83% had awareness regarding the quarantine time; 82% used face masks whenever they left their homes; 98% were aware of the origin of the disease; fever and difficulty of breathing were correctly described as most common symptoms by 70%; 69% correctly stated that the disease can be transferred by touching each other or infected surfaces; and 21% stated that transmission of disease could be through running nose and aerosol.

**Figure 2.**
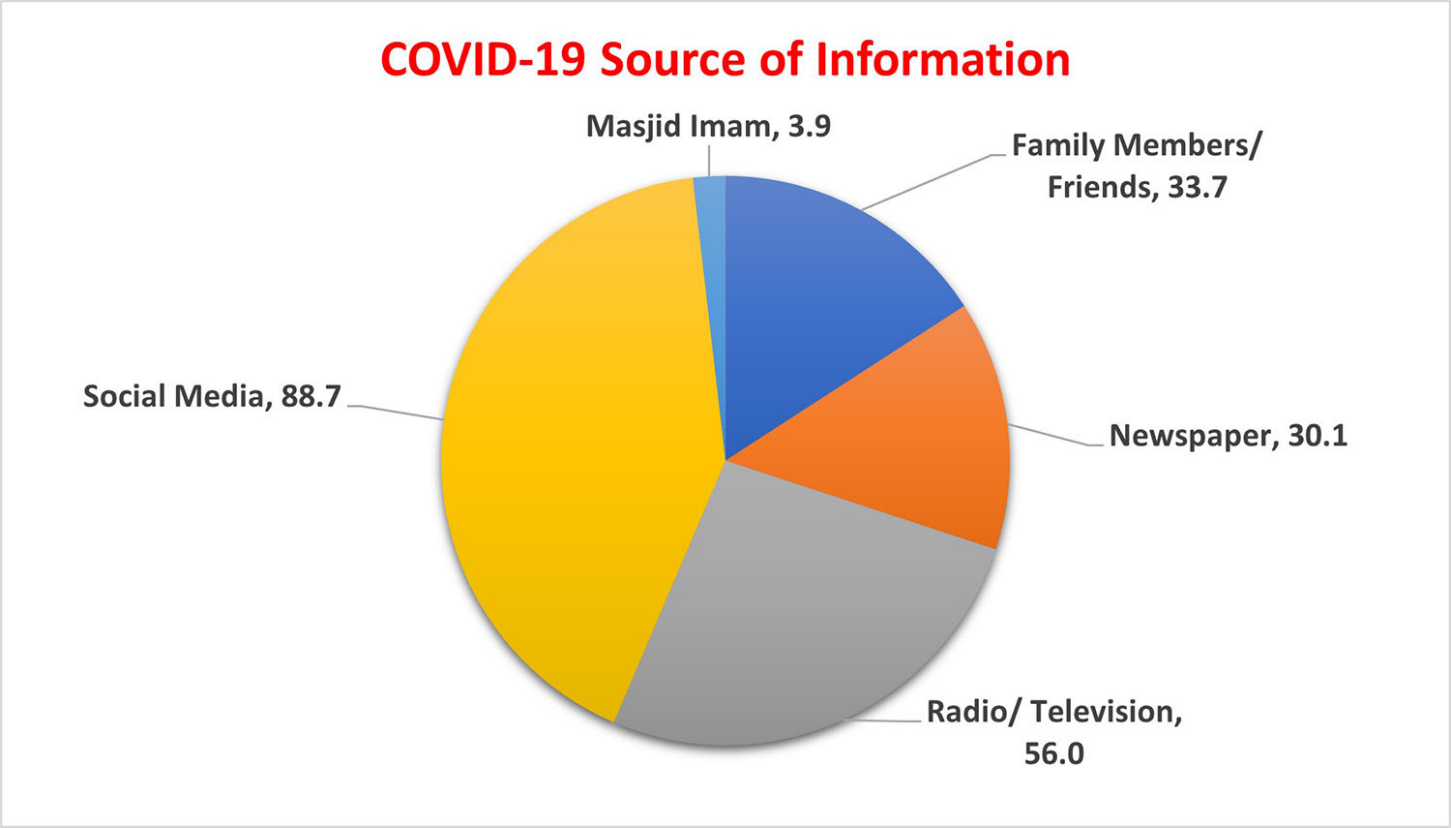
Participants source of information on COVID-19.

Comparisons of mean knowledge score with demographic characteristics are shown in Table 2. There was no significant difference between male and female participant’s knowledge score (9.45 ± 2.8 and 9.51 ± 2.95, respectively; p = 0.86). Age showed significant variation with a higher mean score of knowledge obtained in the 30-45 age group (10.01 ± 2.46) and lower mean score obtained from younger individuals (9.04 ± 2.8) (p = 0.040). Postgraduate participants showed highest mean score (10.5 ± 2.88), although comparison with non-postgraduate groups was not significant (p-value 0.137). Those working from home have significantly higher mean score (9.78 ± 2.8) as compared to those who were not employed (8.02 ± 1.8) (p = 0.029). Participants with seasonal or medicinal allergies showed higher knowledge score (10.44 ± 2.8) than disease free participants, but the difference was not statistically significant (p = 0.32). Similarly, arthritis patients have higher knowledge of COVID-19 as compared to others but not significant (12 ± 3.66). The most stated source of getting information regarding COVID-19 was social media (89%), the television and radio (56%), followed by family friends (34%) and print media (newspapers; 30%) ( Figure 2).

**Table 2.** Comparisons of mean knowledge score with demographic characteristics.

Demographic characteristics	Mean	SD	p-value
**Gender**			
Male	9.45	2.834	0.86
Female	9.51	2.958	
**Age in years**			
15-30	9.04	2.881	0.04
30-45	10.01	2.646	
45-60	9.77	3.320	
**Education level**			
Primary/Secondary	9.75	2.8	0.137
College graduate	9.06	2.91	
Postgraduate	10.5	2.88	
Doctorate	8.7	2.71	
**Working status**			
Yes (working from home)	9.78	2.879	0.029
No	8.02	2.850	
**Monthly income**			
Less than 30K	9.32	2.83	0.627
30K to 50K	9.44	2.6	
More than 50K	10.1	3.62	
**Smoking status**			
Yes	9.7	2.90	0.318
No	9.4	2.86	
Occasional smoker	9.75	3.07	
Past smoker	11.30	2.53	
**Health background**			
*Respiratory disease or any allergy*			
Yes	9.74	2.944	0.32
No	9.35	2.868	
Seasonal/medicinal allergy	10.44	2.80	
*Underlying medical condition/systemic diseases*			
Fit and healthy	9.42	2.678	0.203
Heart disease	9.00	0.000	
Hypertension	8.75	3.130	
Arthritis	12.00	3.633	
Any other disease	9.67	3.541	

Table 3 reveals the hand hygiene awareness of the participants. Almost all of the participants (1030; 92%) were washing their hands multiple times a day). Face washing as a part of Wadu (ablution before prayers) was observed by 504 (45%) individuals and 39% washed their face multiple times. Plain soap and antiseptic solution was found as most popular type of soap used for washing hands (426; 38% and 414; 37%, respectively). More than half (660; 59%) were aware regarding the hand washing technique.

**Table 3.** Awareness level of participants regarding maintenance of personal hygiene.

	n	%
**No. of times you wash your hands**		
Once a day	12	1.0
Twice a day	34	3.0
Multiple times a day	1030	92.0
Without soap (Wudu)	44	4.0
**No. of times you wash your face**		
Once a day	68	6
Twice a day	112	10
Multiple times a day	436	39
Five times Wudu (Wuzu)	504	45
**Kind of soap used for washing**		
Plain soap	426	38
Antiseptic solution	414	37
Antibacterial soap	12	1
Cloth washing soap	12	1
Liquid soap	258	23
**Duration of washing**		
10 Sec	134	12
10-20 Sec	258	23
More than 20 Sec	68	6
1 minute	4	0.4
**Aware of hand washing technique**		
Yes	660	59
No	22	2
May Be	34	3

Those who wash their hands twice a day showed significantly higher knowledge scores (11.0 ± 3.6; p = 0.0001; Table 4). Those who washed their face multiple times also have significantly higher knowledge of COVID-19 (11.14 ± 2.7; p = 0.005). Duration of hand washing and awareness of the technique did not show any significant relation with knowledge score (p = 0.369 and p = 0.286, respectively).

**Table 4.** Knowledge score with hygiene awareness.

	Mean	SD+-	p-value
**No. of times you wash your hands**			
Once a day	9.00	3.606	0.0001
Twice a day	11.00	2.872	
Multiple times a day	9.53	2.812	
Without soap (Wudu)	8.56	2.128	
**No. of times you wash your face**			
Once a day	9.73	2.886	0.005
Twice a day	9.34	2.380	
Multiple times a day	11.14	2.795	
Five times Wudu (Wuzu) (Ablution)	9.42	2.752	
**Kind of soap used for washing**			
Plain soap	9.72	2.773	0.001
Antiseptic solution	9.38	2.812	
Antibacterial soap	10.50	3.416	
Liquid soap	9.44	2.856	
**Duration of washing**			
10 sec	8.79	2.750	0.369
10-20 sec	9.79	3.234	
More than 20 sec	9.78	3.040	
1 minute	7.00	0.000	
**Aware of hand washing technique**			
Yes	9.85	2.910	0.286
No	8.67	2.160	
May be	8.56	3.468	

In total 45% of the participants were afraid of COVID-19 and 47% would like to have more information about the virus and its development. The majority of participants (65%) agreed that the virus is deadly and is life threatening. Only 196 (18%) of the participants had a friend or relative infected with the disease ( Table 5).

**Table 5.** Frequency of the learning attitude of the individuals.

	n	%
**Like to have more information about COVID-19**		
Yes	532	48
No	480	43
Don't know	96	9
**Tired of listening about COVID-19**		
Yes	700	63
No	100	9
I don't know	60	5
Absolutely	160	14
Not Sure	84	8
**COVID-19 is a real-life threatening disease**		
Agree	724	65
Not agree	76	7
I don't know	64	6
May be	240	21
**your friend or relative had COVID-19**		
Yes	196	18
No	872	78
Don't know	40	4

Multiple liner regression showed that male gender (vs female, β – 0.319) and respondents earning between 30K to 50K (vs 10K-30K, β – 0.184) presented low knowledge score, whereas 30-45 year olds (vs 15-30 year, β – 1.148, P = 0.003) presented significantly highest knowledge scores. Age increases and knowledge score decreases β – 1.076 ( Table 6).

**Table 6.** Univariate and multivariate regression.

Variables	Partial regression coefficients		Standardized coefficients	t	Sig.
	*B*	*Std. Error*	*Beta*		
(Constant)	8.451	0.496		17.024	0.000 *
Gender (male vs Female	−0.319	0.358	−0.055	−0.892	0.373
Job (yes vs No)	0.825	0.444	0.140	1.858	0.064
Income (30K to 50K vs 10K-30K)	−0.184	0.458	−0.028	-0.402	0.688
Income (More than 50K vs 15K-30K)	0.395	0.475	0.066	0.832	0.406
Age (30-45 vs 15-30 years)	1.148	0.377	0.199	3.044	0.003 *
Age (45 and above vs 15-30 years)	1.076	0.831	0.081	1.295	0.196

*Significant at 0.05, F = 2.575, p = 0.019, R ^2^ = 0.054, R ^2^ adjusted showed that regression had 3% predictive accuracy.

## Discussion

To the best of author’s knowledge this is among the first studies evaluating the awareness, knowledge and preventive practice of the public in Pakistan during the pandemic. Overall, 70% of the public studied gave the correct answers related to knowledge of COVID-19. These results are in agreement with a study conducted in India where a knowledge rate of 74% has been reported, ^15^ while it is comparatively less when compared with study conducted in China (90%). ^16^ However these findings are higher when compared to the response received by a study conducted in Jordan 40%. ^17^ The reason for such a high response rate within China may be due to difference in time and circumstance in which the study was conducted. On the contrary, in the current study the high response can be attributed to the campaigns related to COVID-19 awareness initiated by the Pakistani government just after the first cases started being reported within neighbouring countries and also possibly due to the fact that many participants had higher education levels that included 42% graduates and 47% postgraduates.

The commonest source of acquiring information related to COVID-19 was through social media (89%), followed by television/radio. Similar results had been revealed by a study conducted in China. ^16^ The majority of participants (83%) knew that the incubation period for coronavirus is 14 days, whereas another study from China reported it as 2.5 days. ^18^ However, the CDC also suggested it can be range from 2 to 14 days. ^19^ Common symptoms of COVID-19 are fever, cough, fatigue and shortness of breath. ^20^ However, elderly people with underlying medical condition, such as hypertension, heart disease, chronic obstructive pulmonary disease coronary patients and those with chronic respiratory disease and frontline health providers are at a greater risk of acquiring the infection. ^21^

In total, 75% of participants from the current study had awareness regarding COVID-19 symptoms, while 82% knew the importance of wearing face masks as preventive measures, and 69% had sufficient knowledge regarding coronavirus being transmitted through different surfaces. Our findings are in line with another study conducted among Pakistani university students, their knowledge about incubation was lower at 53%, whereas the awareness with common symptoms was higher (93.7%). ^22^ The transmission of COVID-19 can occur through direct or indirect close contact with the infected person or through discharge of saliva, and respiratory emissions or droplets which can be emitted from infected persons during coughing, sneezing or spiting. ^23^^,^^24^ Respiratory droplets normally are greater than 5-10 μm and aerosol droplets are less than 5 μm in diameter; they can be transferred to any individual when a normal person comes in close contact with the infected person. ^25^ Aerosol transmission awareness was noted to be significantly lower in this study (21%), which is in agreement with a study conducted in India. ^15^ Hence, there is a need to increase the awareness level of people regarding the modes of transmission of COVID-19 and to acknowledge its significance.

Another study from China showed that 93% of study participants agreed that COVID-19 transmission can be prevented by thoroughly washing hands with soap. ^26^ These facts agree with the findings of the study; 92% of the participants washed their hands multiple times a day. As a preventive practice using antibacterial soap was significantly associated with knowledge score (10.5 ± 3.41; p < 0.001) which is similar to a study conducted in Jordan where 87% adopted hand washing as a preventive measure. ^17^ Almost 60% of participants in the present study were aware of hand washing technique. A former study concluded that washing hands with soap and water is enough to reduce the risk of viral infections and when it is practiced with the recommended protocol of hand washing technique it reduces the rate of transmission of COVID-19. ^27^

The findings of the current study suggested that the majority of participants had good learning attitude towards COVID-19; however lower knowledge level and negative attitudes were also recorded. A total of 63% individuals were tired of listening and did not want further information about the virus. One reason may be that majority (78%) had no relative or friend who had been infected with the virus. Additional educational campaigns are needed for the general public for further guiding them regarding the mode of transmission, isolation period and the different adoptive preventive strategies (like social distancing, avoiding handshakes, wearing masks and gloves), along with the risk of personal and family infection with COVID-19. The strength of the current study was that it was a nationwide survey with a large sample, and that the research was conducted during the surging stage of the COVID-19 pandemic.

### Limitations

The study is not a true representative sample of Pakistan’s general population. It’s a convenience sample through social media, which has its own limitations. Social media statistics are dynamic and can change according to its popularity and due to specific group of users being educated and having access to that information. This is the reason most of the participants were graduates and above.

## Conclusion

It can be concluded from the current study that female participants had a higher level of education, and more awareness regarding the coronavirus. The majority of the participants followed a proper hand washing regime and washed their face. It also highlighted the power of social media as the source of information. Further knowledge and awareness should be promoted. Future work should focus on a larger, national representative sample population.

## Data availability

### Underlying data

Harvard Dataverse: General Public Awareness, Knowledge and Attitude toward COVID-19 infection and prevention: A cross sectional study from Pakistan, https://doi.org/10.7910/DVN/1K5LDD. ^28^

### Extended data

Harvard Dataverse: General Public Awareness, Knowledge and Attitude toward COVID-19 infection and prevention: A cross sectional study from Pakistan, https://doi.org/10.7910/DVN/1K5LDD. ^28^

This project contains the following extended data:

- Questionnaire

Data are available under the terms of the Creative Commons Zero “No rights reserved” data waiver (CC0 1.0 Public domain dedication).
