# Supplementary material for: General public awareness, knowledge and attitude toward COVID-19 infection and prevention: a cross-sectional study from Pakistan
Source: F1000Res. 2023 Jun 5;10:946. Originally published 2021 Sep 21. [Version 2] doi: 10.12688/f1000research.52692.2 (PMC10285421; doi:10.12688/f1000research.52692.2)
Supplement: Peer review history

# Peer review: Reviewer response for version 2

**DOI:** 10.5256/f1000research.146374.r176785

**Published:** 2023-06-06

**Authors:** Mohmed Isaqali Karobari

**Affiliations:** 1 Department of Conservative Dentistry and Endodontics, Saveetha Dental College and Hospitals, Saveetha Institute of Medical and Technical Sciences, Saveetha University, Chennai, India; 2 Conservative Dentistry Unit, School of Dental Sciences, Universiti Sains Malaysia, Kota Bharu, Malaysia; 3 Department of Restorative Dentistry & Endodontics, Faculty of Dentistry, University of Puthisastra, Phnom Penh, 12211, Cambodia

Dear Authors,

 The authors have addressed all the comments and the manuscript has much improved. I would like to congratulate the authors and wish them all the very best for future endeavors.

 Best regards and keep well

Is the work clearly and accurately presented and does it cite the current literature?

Yes

If applicable, is the statistical analysis and its interpretation appropriate?

Yes

Are all the source data underlying the results available to ensure full reproducibility?

Yes

Is the study design appropriate and is the work technically sound?

Yes

Are the conclusions drawn adequately supported by the results?

Yes

Are sufficient details of methods and analysis provided to allow replication by others?

Yes

Reviewer Expertise:

Dentistry, Endodontics, Restorative, Dental education, COVID

I confirm that I have read this submission and believe that I have an appropriate level of expertise to confirm that it is of an acceptable scientific standard.

# Peer review: Reviewer response for version 1

**DOI:** 10.5256/f1000research.56002.r94965

**Published:** 2021-10-15

**Authors:** Anand Marya

**Affiliations:** 1 Department of Orthodontics, Faculty of Dentistry, University of Puthisastra, Phnom Penh, Cambodia; 2 Department of Orthodontics, Saveetha Dental College & Hospitals, Saveetha Institute of Medical and Technical Sciences (SIMATS), Saveetha Univeristy, Chennai, Tamil Nadu, India

I would like to thank the editor for inviting me to review this manuscript. I would also like to compliment the authors on a well-conducted study that is very relevant during the current pandemic.

 The authors have made an effort to conduct a KAP study on the public in Pakistan to evaluate their response towards COVID-19. They conducted a survey using 1120 people for data collection and also ran the Student's t-test and ANOVA after the data collection. They concluded that female participants in the study had a higher awareness regarding the SARS-CoV2 pandemic and also found out that people generally had good awareness about hand hygiene methods.

 I only have a few suggestions to help improve the quality of the paper:

- Please include a power of the sample part/discussion. Although the investigators have taken a sample of 1120, it would be a good idea to run a power of the sample test to demonstrate the population this sample would represent.
- Please include a few points of collecting information online in the limitations section. Each study has its own limitations and online surveys generally have their own limitations and bias. Kindly elaborate on this in a separate section.

Also, you may find the following studies helpful to add to your discussion. The reason for using these studies is they are relevant to your paper and also would help you add to the discussion and limitations sections as both are online surveys on very closely related topics that have been published in high-impact journals. 

1. Marya *et al.*, 2021 ^1^: This study focuses on what measures are being taken to curtail the spread of COVID-19 by dentists to ensure no contact spread or cross-contamination occurs.
2. Albargi *et al.,* 2021 ^2^: This is a very important paper that highlights the attitude of the patients in many situations where they do not divulge proper information regarding their COVID-19 status. Again, this paper is very important in demonstrating the attitude of the general public towards COVID-19.

Is the work clearly and accurately presented and does it cite the current literature?

Yes

If applicable, is the statistical analysis and its interpretation appropriate?

Yes

Are all the source data underlying the results available to ensure full reproducibility?

Yes

Is the study design appropriate and is the work technically sound?

Yes

Are the conclusions drawn adequately supported by the results?

Yes

Are sufficient details of methods and analysis provided to allow replication by others?

Yes

Reviewer Expertise:

Orthodontics, oral health, dental public health

I confirm that I have read this submission and believe that I have an appropriate level of expertise to confirm that it is of an acceptable scientific standard.

## References

[1] : Risk Perception of SARS-CoV-2 Infection and Implementation of Various Protective Measures by Dentists Across Various Countries. *Int J Environ Res Public Health* .2021;18(11) : 10.3390/ijerph18115848 10.3390/ijerph18115848 34072456PMC8199051

[2] : Analysis of the reliability of patient COVID-19 screening data compared against their actual test data. *Sci Prog* .104(3) : 10.1177/00368504211042980 368504211042980 10.1177/00368504211042980 34541957PMC10461374

# Response

**Published:** 2023-03-30

**Authors:** Faraz Farooqi

**Affiliations:** Imam Abdulrahman Bin Faisal University, Saudi Arabia

I only have a few suggestions to help improve the quality of the paper:

 Please include a power of the sample part/discussion. Although the investigators have taken a sample of 1120, it would be a good idea to run a power of the sample test to demonstrate the population this sample would represent.

*Response: Power calculation has been included.*

 Please include a few points of collecting information online in the limitations section. Each study has its limitations and online surveys generally have their own limitations and bias. Kindly elaborate on this in a separate section.

*Response: has been elaborated.*

 Also, you may find the following studies helpful to add to your discussion. The reason for using these studies is they are relevant to your paper and also would help you add to the discussion and limitations sections as both are online surveys on very closely related topics that have been published in high-impact journals. 

 Marya et al., 2021 1: This study focuses on what measures are being taken to curtail the spread of COVID-19 by dentists to ensure no contact spread or cross-contamination occurs.

 Albargi et al., 2021 2: This is a very important paper that highlights the attitude of the patients in many situations where they do not divulge proper information regarding their COVID-19 status. Again, this paper is very important in demonstrating the attitude of the general public toward COVID-19.

*Response: the suggested articles have been added.*

# Peer review: Reviewer response for version 1

**DOI:** 10.5256/f1000research.56002.r96322

**Published:** 2021-10-14

**Authors:** Mohmed Isaqali Karobari

**Affiliations:** 1 Department of Conservative Dentistry and Endodontics, Saveetha Dental College and Hospitals, Saveetha Institute of Medical and Technical Sciences, Saveetha University, Chennai, India; 2 Conservative Dentistry Unit, School of Dental Sciences, Universiti Sains Malaysia, Kota Bharu, Malaysia; 3 Department of Restorative Dentistry & Endodontics, Faculty of Dentistry, University of Puthisastra, Phnom Penh, 12211, Cambodia

The manuscript under review attempts to evaluate the general public awareness, knowledge, and attitude towards COVID-19 infection and prevention amongst the Pakistani population. In general, the manuscript captures the details of the study design and implementation of the project. All the sections of the manuscript are well written and concluded, although several limitations exist, and it has been presented in the manuscript. The study is of sound design and clear practical and clinical interest, and I suggest accepting this article with minor revisions.

 Following are the minor comments:

**Abstract:**

- *Background: *The authors have mentioned aims; either change the subheading to aims and add another subheading as background or write an introduction of the study.
- *Results:* Kindly mention the statistical significance.

**Methods:**

- Is the study conducted in a Pakistani subpopulation or a Pakistani population? 

**Results:**

- The description in the results section is a duplication of tables and figures. Kindly avoid the repetition of data that is already presented in the tables and figures.

**Discussion:**

- Kindly avoid writing results in the discussion; the authors can mention the significant difference.
- Write future recommendations.

Is the work clearly and accurately presented and does it cite the current literature?

Yes

If applicable, is the statistical analysis and its interpretation appropriate?

Yes

Are all the source data underlying the results available to ensure full reproducibility?

Yes

Is the study design appropriate and is the work technically sound?

Yes

Are the conclusions drawn adequately supported by the results?

Yes

Are sufficient details of methods and analysis provided to allow replication by others?

Yes

Reviewer Expertise:

Dentistry, Endodontics, Restorative, Dental education, COVID

I confirm that I have read this submission and believe that I have an appropriate level of expertise to confirm that it is of an acceptable scientific standard, however I have significant reservations, as outlined above.

# Response

**Published:** 2023-03-30

**Authors:** Faraz Farooqi

**Affiliations:** Imam Abdulrahman Bin Faisal University, Saudi Arabia

Abstract:

 Background: The authors have mentioned aims; either change the subheading to aims and add another subheading as background or write an introduction of the study.

*Response: separate section of background and aims has been added in the abstract section.*

**

 Results: Kindly mention the statistical significance.

*Response: has been added.*

 Methods:

 Is the study conducted in a Pakistani subpopulation or a Pakistani population? 

*Response: the study was conducted on the Pakistani population, not a subpopulation.*

 Results:

 The description in the results section is a duplication of tables and figures. Kindly avoid the repetition of data that is already presented in the tables and figures.

*Response: has been corrected.*

 Discussion:

 Kindly avoid writing results in the discussion; the authors can mention the significant difference.

*Response: has been corrected.*

**

 Write future recommendations.

*Response: Has been added.*
